# The methylerythritol phosphate pathway as an oxidative stress sense and response system

**DOI:** 10.1038/s41467-024-49483-8

**Published:** 2024-06-21

**Authors:** Jordi Perez-Gil, James Behrendorff, Andrew Douw, Claudia E. Vickers

**Affiliations:** 1 ARC Centre of Excellence in Synthetic Biology, Queensland University of Technology, Brisbane, QLD 4000 Australia; 2 Centre for Agriculture and the Bioeconomy, Queensland University of Technology, Brisbane, QLD 4000 Australia; 3 School of Environmental and Biological Science, Queensland University of Technology, Brisbane, QLD 4001 Australia; 4 School of Chemistry and Molecular Biosciences, The University of Queensland, St Lucia, QLD 4072 Australia; 5 BioBuilt Solutions, Corinda, QLD 4075 Australia

**Keywords:** Biochemistry, Chemical biology

## Abstract

The methylerythritol phosphate (MEP) pathway is responsible for biosynthesis of the precursors of isoprenoid compounds in eubacteria and plastids. It is a metabolic alternative to the well-known mevalonate pathway for isoprenoid production found in archaea and eukaryotes. Recently, a role for the MEP pathway in oxidative stress detection, signalling, and response has been identified. This role is executed in part through the unusual cyclic intermediate, methylerythritol cyclodiphosphate (MEcDP). We postulate that this response is triggered through the oxygen sensitivity of the MEP pathway’s terminal iron-sulfur (Fe-S) cluster enzymes. MEcDP is the substrate of IspG, the first Fe-S cluster enzyme in the pathway; it accumulates under oxidative stress conditions and acts as a signalling molecule. It may also act as an antioxidant. Furthermore, evidence is emerging for a broader and highly nuanced role of the MEP pathway in oxidative stress responses, implemented through a complex system of differential regulation and sensitivity at numerous nodes in the pathway. Here, we explore the evidence for such a role (including the contribution of the Fe-S cluster enzymes and different pathway metabolites, especially MEcDP), the evolutionary implications, and the many questions remaining about the behaviour of the MEP pathway in the presence of oxidative stress.

## Abstract (web summary)

A new role for the MEP pathway has emerged in oxidative stress detection, signalling, and response. The authors compile all the evidence and postulate that this sensing ability and response occurs through the oxygen sensitivity of its iron-sulphur cluster enzymes.

## Introduction

Production of reactive oxygen species (ROS) is an unavoidable consequence of metabolism in an aerobic world, and ROS play important roles in normal physiological processes (e.g. photosynthesis). However, ROS in metabolism is a double-edged sword. Under normal biological conditions, cells maintain a delicate balance with metabolic ROS. Under stress, this balance is overcome and over-accumulation of ROS, including superoxide (O_2_^**-**^), hydroxyl radical (OH**·**), hydrogen peroxide (H_2_O_2_), and singlet oxygen (^1^O_2_), occurs^1^. This causes damaging oxidation of biological components (nucleic acids, proteins, and lipids). This in turn results in oxidative stress, which is implicated in human, plant, and animal disease^2,3^. To manage cellular ROS, cells have evolved a variety of mechanisms to sense ROS levels and respond to accumulation by production of antioxidant metabolites and enzymes that neutralise ROS and protect biological components^1,4^.

**Fig. 1:**
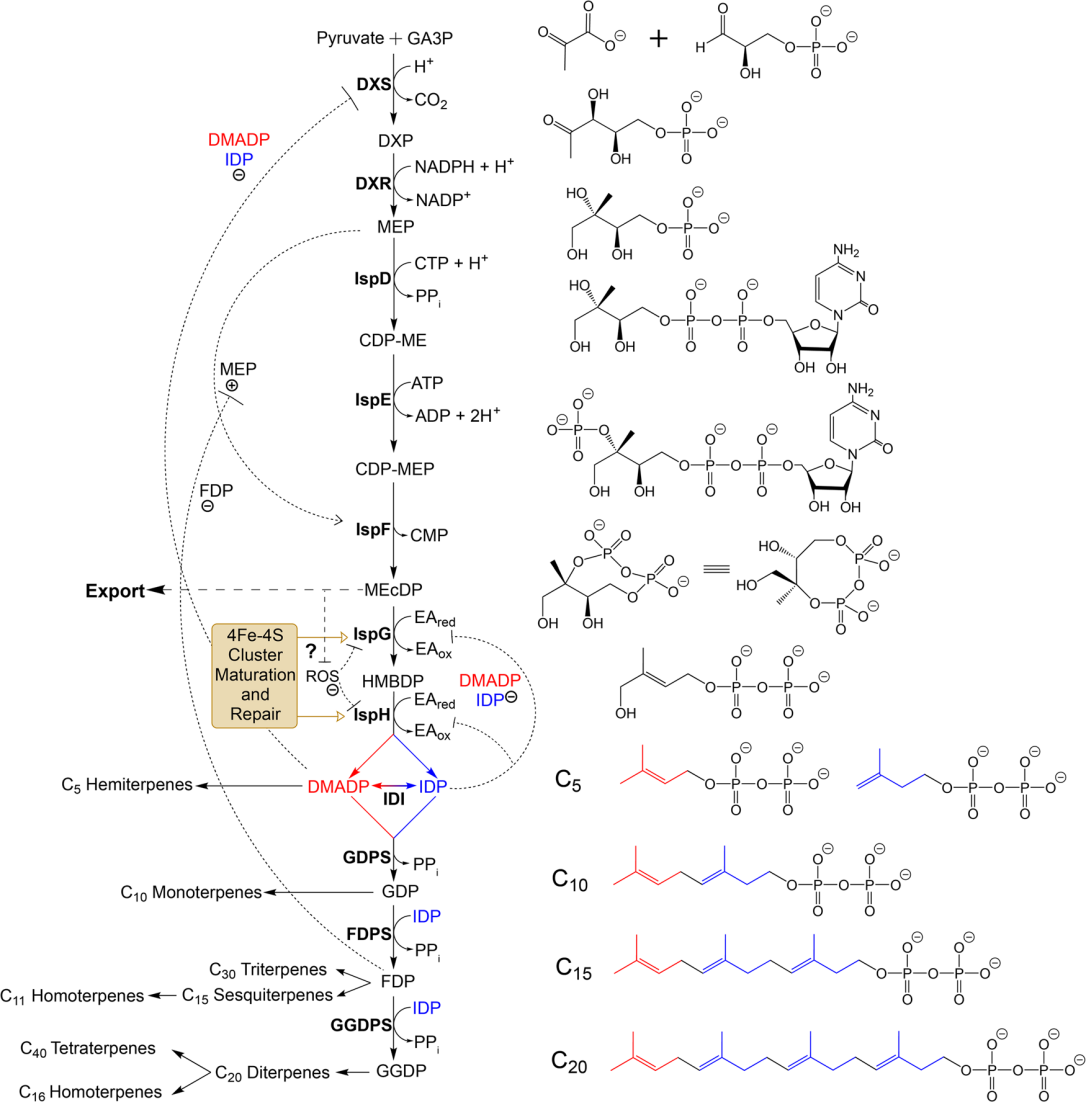
The methylerythritol phosphate (MEP) pathway and downstream prenyl phosphate pathway, including cofactors, prosthetic groups, negative and positive feedback, feed forward regulation, and product classes. Nomenclature follows that identified by Phillips et al.^26^ for microbial systems. C_5_ derivatives are referred to as hemiterpenes and include isoprene and other highly volatile biogenic gases. C_10_ derivatives are referred to as monoterpenes and are also highly volatile; they tend to have signalling and defence functions. C_15_ derivatives are called sesquiterpenes; there is a C_11_ class of sesquiterpenes derivatives called homoterpenes, and a C_30_ class, composed of two FDP moieties, called triterpenes (sterols are an example of triterpenes). Many sesquiterpene compounds are also quite volatile; others are non-volatile and form parts of important molecules with additional prosthetic groups, such as haemes and quinones. C_20_ derivatives are known as diterpenes, and include important prosthetic groups for chlorophylls, tocopherols, and phyloquinones. There is also C_16_ class of sesquiterpenes derivatives that is also called homoterpenes. The tetraterpene class is formed from two GGDP moieties (C_40_) and includes the carotenoid pigments and the plant hormone abscisic acid (ABA). Chemical structures of the MEP pathway are shown in black, with the exception of IDP which is shown in blue, and DMADP which is shown in red. Carbons contributed from IDP and DMADP are represented in their respective colours in the higher-order downstream prenyl phosphate structures. Enzyme abbreviations: DXS 1-deoxyxylulose-5-phosphate synthase, DXR 1-deoxyxylulose-5-phosphate reductoisomerase (a.k.a. IspC), IspD 4-diphosphocytidyl-2*-C-*methyl-d-erythritol synthase (a.k.a. MCT), IspE 4-diphosphocytidyl-2-*C*-methyl-d-erythritol 2-phosphate kinase (a.k.a. CMK), IspF 2-*C*-methyl-d-erythritol 2,4-cyclodiphosphate synthase (a.k.a. MDS), IspG 1-hydroxy-2-methyl-2-(*E*)-butenyl 4-diphosphate synthase (a.k.a. HDS), IspH 1-hydroxy-2-methyl-2-(*E*)-butenyl 4-diphosphate reductase (a.k.a. HDR), IDI isopentenyl diphosphate isomerase. Metabolite abbreviations: GA3P Glyceraldehyde 3-phosphate, DXP 1-deoxyxylulose 5-phosphate, MEP 2*-C-*methyl-d-erythritol 4-phosphate, CDP-ME 4-diphosphocytidyl-2*-C-*methyl-d-erythritol, CDP-MEP 4-diphosphocytidyl-2*-C-*methyl-d-erythritol 2-phosphate, MEcDP 2*-C-*methyl-d-erythritol 2,4-cyclodiphosphate, HMBDP 1-hydroxy-2-methyl-2-(*E*)-butenyl 4-diphosphate, DMADP dimethylallyl diphosphate (see Box 1 part b), IDP isoprenyl diphosphate (see Box 1 part b), GDP geranyl diphosphate, FDP farnesyl diphosphate, GGDP geranylgeranyl diphosphate, PP_i_ inorganic diphosphate, ATP adenosine triphosphate, ADP adenosine diphosphate, CTP cytidine triphosphate, CMP cytidine monophosphate, ROS reactive oxygen species, EA_red_ reduced electron acceptor, EA_ox_ oxidised electron acceptor, NADP^+^ oxidised nicotinamide adenine dinucleotide phosphate, NADPH reduced nicotinamide adenine dinucleotide phosphate.

The methylerythritol phosphate (MEP) pathway (Fig. 1) is found in most bacteria, in some lower eukaryotes (e.g. plastidial apicomplexans), and in plant plastids^5^. In these organisms/organelles, it replaces the mevalonate pathway for production of isoprenoids (terpenoids/terpenes). Isoprenoids are the largest class of natural products and fulfil a host of essential functions in all free-living organisms. Many of these functions are related to management of oxidative stress^6^, for example, the volatile hydrocarbon isoprene, vitamin E (tocopherol), and carotenoids (e.g. β-carotene and astaxanthin) all act in oxidative stress responses.

The MEP pathway contains two iron-sulfur (Fe-S) cluster enzymes at the ultimate and penultimate steps of the pathway (Fig. 1). Fe-S cluster proteins are ancient and ubiquitous proteins, and are postulated to have evolved within some of the first life on Earth^7^. Going further back, Fe-S cluster chemistry may have played a role in the origin of life at proto-cell/mineral interfaces^7,8^. Comprised of iron (Fe^2+/3+^) connected by inorganic sulfide (S^2-^) (most commonly in clusters of 2Fe-2S and 4Fe-4S), Fe-S clusters are prosthetic groups that are coordinated to the protein via amino acid sidechains, most commonly the thiolate of cysteine^9^. These enzymes catalyse key steps controlling metabolic flux in the MEP pathway, as well as being prospective drug targets for the development of antimicrobial agents and herbicides^10^.

Here we explore the history and relevance of the MEP pathway and its Fe-S cluster enzymes in the context of oxidative stress. In particular we explore the oxygen sensitivity of these enzymes with respect to MEP pathway regulation, the overall function of the MEP pathway, and the behaviour of the intermediate metabolite 2*-C-*methyl-d-erythritol-2,4-cyclodiphosphate (MEcDP, also known as MEC^11^ or MEcPP; see Box 1 part a), which plays a key role in stress responses.

Box 1 Notes on nomenclature a Historically, MEcPP (for methylerythritol pyrophosphate) has been the most common nomenclature. However, the field is now moving towards the more modern use of ‘diphosphate’ instead of the more archaic ‘pyrophosphate’, and hence, use of MEcDP rather than MEcPP. We have therefore chosen to use MEcDP, and are using the ‘DP’ for ‘diphosphate’ throughout in our nomenclature.

b As noted above, the field is now moving towards the more modern use of ‘diphosphate’ rather than the archaic ‘pyrophosphate’. We have therefore chosen to use DMADP and IDP for DMAPP and IPP.

c There is significant confusion in the literature regarding the nomenclature of the MEP pathway, as more than one name is often published for enzymes in the pathway. Phillips et al.^26^ proposed a unified nomenclature based on the system used by plant biologists, however this system has not gained much traction in fields outside of plant biology/biotechnology. Most commonly, enzymes in the MEP pathway are referred to by their ‘Isp’ monikers, except for DXR, DXS, and IDI, which for historical reasons commonly use full enzyme name abbreviation nomenclature.

### Isoprenoids and the methylerythritol phosphate pathway

Two biosynthetic pathways produce the two universal building blocks for isoprenoids, isopentenyl diphosphate (IDP) and dimethylallyl diphosphate (DMADP; see Box 1 part b): the mevalonate (MVA) pathway (predominantly found in animals, fungi, archaea, some bacteria, and the plant cell cytosol) and the MEP pathway (found in most bacteria, some plastidial parasites, and plant chloroplasts)^6^. The pathways follow distinct and unrelated chemistry, but produce identical terminal products, IDP and DMADP. The MVA pathway initiates from central carbon metabolism by condensation of two acetyl-CoA molecules, while the MEP pathway initiates from the condensation of pyruvate and glyceraldehyde 3-phosphate (GA3P; Fig. 1).

Downstream from IDP and DMADP, a series of condensation reactions using DMADP as the allylic primer deliver a series of increasing (by 5-carbons) chain-length phosphorylated precursors, which are then dephosphorylated and modified using the full range of available biochemical activities to deliver a massive array of different molecules (86,052 identified to date (dnp.chemnetbase.com); Fig. 1). This shared component of the MVA and MEP pathway is referred to as prenyl phosphate metabolism.

Their remarkable chemical diversity allows isoprenoids to deliver on diverse physiological roles, including as components of crucial quinones in the electron transport chain, pigments such as chlorophyll and carotenoids, and membrane sterols such as cholesterol (the precursor to all animal steroids)^12–15^. Isoprenoids also have functions as secondary metabolites for cellular signalling, hormones, defence against herbivores, stress responses, and mutualistic interactions (e.g. root-based fungi), among many others^6,13,16,17^. These functions have predominantly been characterised in plants.

The MEP pathway possesses some promising targets for the biotechnological and chemical industries. It is essential for primary isoprenoid metabolism in most bacteria and Apicomplexa parasites, as well as being integral to the production of chlorophyll and related compounds in plants^18^. This positions it as an interesting target for herbicides and antimicrobials, as the pathway is absent in humans and other animals^10^. The chemical diversity and biological functionality of isoprenoids also delivers a wide range of industrial uses, with applications including pharmaceuticals, nutraceuticals, fragrances, flavours, colourants, agricultural chemicals, biofuels, rubbers, resins, solvents, and much more^10,19^. However, economic recovery from their predominantly plant hosts is commonly not possible due to low levels, variability in production, or complexity of extraction/purification. Chemical synthesis of these often-complex molecules may be challenging or impossible, or simply too expensive. Metabolic engineering of microbial hosts (e.g. *Escherichia coli* and *Saccharomyces cerevisiae*) can allow for the high-titre production of numerous isoprenoids to overcome these constraints on industrial translation of these molecules^19,20^. While this has primarily been explored using the MVA pathway (either native or heterologously expressed) in microbial hosts, the MEP pathway possesses a higher potential for isoprenoid production over the MVA pathway, namely, a higher theoretical carbon yield in the context of glycolysis (30.2% for the MEP pathway on glucose compared to 25.2% for the MVA pathway)^21^. Genome scale modelling in the context of *S. cerevisiae* metabolism showed that 1 mol glucose could yield a maximum of 0.21 and 0.24 mol farnesyl diphosphate (FDP) from the MVA and MEP pathways, respectively^22^. Yields of isoprenoid compounds via the MVA and MEP pathways differ for alternative modes of carbon fixation such as oxygenic photosynthesis, the Wood-Ljungdahl pathway, and various synthetic carbon fixation cycles including non-oxidative glycolysis^23^ and the THETA cycle^24^, but exploitation of the MEP pathway for isoprenoid production remains attractive due to the continuing dependence of industrial fermentation on glycolytic host organisms. Utilisation of the MEP pathway for biochemical synthesis has been hindered by its extremely complex regulation and its oxygen sensitivity^25^ (Fig. 1). This sensitivity is conferred by the two terminal enzymes of the MEP pathway, 1-hydroxy-2-methyl-2-(*E*)-butenyl 4-diphosphate (HMBDP) synthase (**IspG**, also known as HDS or gpcE^26^; see Box 1 part c) and 1-hydroxy-2-methyl-2-(*E*)-butenyl 4-diphosphate reductase (**IspH**, also known as HDR, LytB, or IDS; see Box 1 part c), which are Fe-S cluster enzymes (Fig. 1). Interestingly, while the archaeal MVA pathway includes oxygen-sensitive Fe-S cluster enzymes^27–30^, the more recently-evolved eukaryotic MVA pathway does not contain Fe-S cluster enzymes.

### Fe-S cluster enzymes

Discovered in the 1960s, Fe-S cluster enzymes are most well-known for facilitating redox reactions through single and double electron transfers over a vast range of redox potentials, from −650 mV to +450 mV^7,31^. Their sensitivity to ROS, coupled with the difficulty of working in anaerobic environments, meant that Fe-S cluster enzymes were poorly characterised until recent decades; indeed, crystallisation often still eludes us for many of them, and many remain poorly characterised^32,33^.

The ubiquity and essentiality of Fe-S clusters throughout all domains of life, as well as the chemical similarity of Fe-S clusters to pre-biotic inorganic minerals, has led to the hypothesis that Fe-S clusters evolved early in the history of life, and may have even pre-dated cellular life^7^. Evolution in an anaerobic world prior to the Great Oxidation Event would also explain their ubiquity despite the sensitivity of Fe-S clusters to molecular oxygen and ROS. This is further corroborated by the great evolutionary lengths undertaken to protect Fe-S cluster proteins from oxidative damage, such as utilising multiple biosynthetic pathways for cluster formation, ROS-scavenging enzymes like catalase and superoxide dismutase, Fe-S cluster repair pathways, and mutation of amino acids around the active site conferring decreased sensitivity^34,35^. 4Fe-4S clusters that are solvent-accessible are particularly prone to oxidative degradation, and in some cases – such as in IspG and IspH – the active site is buried as a result of a conformational change of the protein following substrate binding, preventing solvent access and protecting the cluster from oxidative damage^36^.

Fe-S cluster chemistry is complex and Fe-S cluster enzymes can be categorised into four different classes based on their biological activities^7,37–39^. The vast majority perform electron transfer as their sole function; some donate sulfur; some behave as sensors for oxidative stress, exploiting the sensitivity of the Fe-S cluster to oxygen; and some perform catalysis (both redox-active and non-redox). Most Fe-S cluster enzymes are limited to one of these activities, although some enzymes utilise atypical, heterogeneous, and/or numerous Fe-S clusters to perform multiple functions in unison^39^.

### The MEP pathway Fe-S cluster enzymes: IspG and IspH

**Fig. 2:**
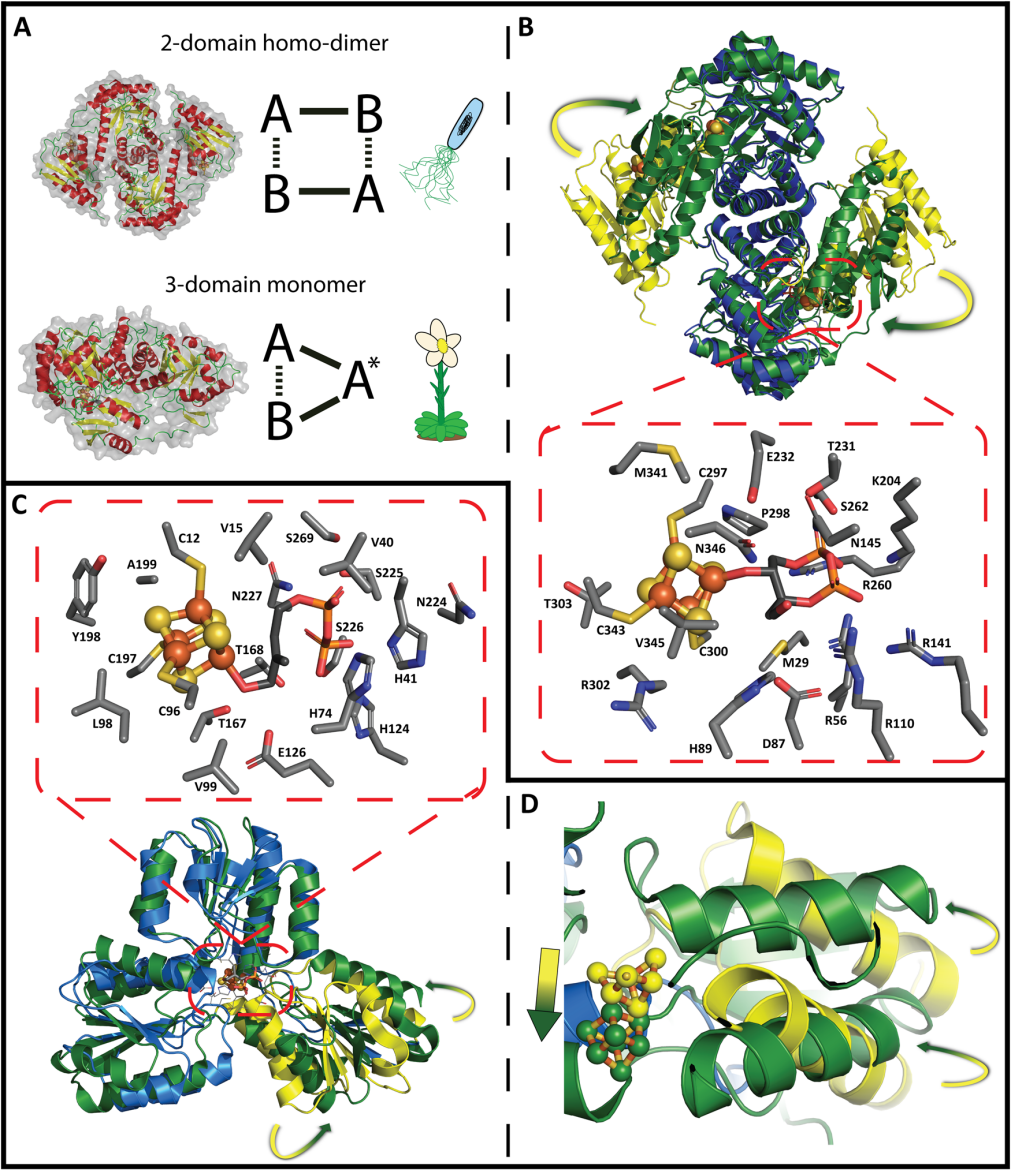
Crystal structures of IspG and IspH showing active site coordination and conformation changes upon binding of the respective ligands. **A** Comparison of two-domain and three-domain IspG, found predominantly in bacteria and plants, respectively. **B** Alignment of *Thermus thermophilus* IspG dimers in substrate-free (2Y0F; blue/yellow) and bound (4G9P; green) forms. The blue regions of the substrate-free dimer indicate no/minimal change after MEcDP binding while the yellow regions indicate major conformational change. This occurs primarily in the C-terminal domains, bringing the 4Fe-4S cluster closer to the MEcDP binding site; another key change involves the movement of the catalytic E232 into the active site (insert). **C** Alignment of substrate-free *Aquifex aeolicus* IspH (3DNF; blue/yellow) and *E. coli* IspH-HMBDP (3KE8; green). As with IspG, the blue regions of IspH indicate minimal change after HMBDP binding, while the yellow region indicates a major confirmational change. This substrate-induced perturbation closes the active site off, helping to protect the Fe-S cluster from oxidative dissociation. **D** Side-profile of the IspH conformational change.

IspG and IspH are both examples of catalytically active Fe-S cluster enzymes. They catalyse a 2e^−^/2H^+^ reductive dehydroxylation and possess some structural and mechanistic similarities. The binding pocket of IspG is composed of positively-charged residues, while the IspH binding pocket is composed of polar residues and extensive hydrogen bonding networks (Fig. 2)^36^. Both binding events are accompanied by a conformational change. In IspG, the C-terminal domain rotates approximately 60 degrees, closing off the active site from bulk solvent^40^. Similarly, IspH rotates one of its three domains 20 degrees to close off the active site^41^. This serves both to protect the Fe-S cluster from oxidative damage and to avoid hydrolysis of the diphosphate group. The active sites of both enzymes incorporate a cubic 4Fe-4S cluster which is coordinated to three cysteine residues, with the fourth Fe site uniquely coordinated for ligand binding (Fig. 2).

## Evidence for a stress-response role of the MEP pathway

When photosynthetic cyanobacteria introduced molecular oxygen into the Earth’s atmosphere 2.4 billion years ago^42^, it reacted with ferrous iron to produce ROS. A highly conserved, tightly regulated response to oxidative stress is therefore found in all organisms today^1,4^. Reactions between oxygen and iron (in the form of Fe-S cluster proteins) also contribute to stress detection and response mechanisms, including the chloroplast sensor kinase (a 3Fe-4S protein that alternately responds to oxidation by oxygen or reduction by the plastoquinone pool to regulate photosystem I gene expression)^43^. Here, we explore several lines of evidence which suggest that the MEP pathway itself (i.e. over and above the distinct antioxidant molecules which are produced via this pathway downstream of prenyl phosphate metabolism) forms an important part of the cellular oxidative stress response system.

### The MEP pathway includes redox-sensitive enzymes and requires reducing power

As noted above, IspG and IspH are both Fe-S cluster enzymes and are therefore sensitive to alteration in redox status, which usually occurs via interaction with ROS^44–46^. Cluster repair is also highly sensitive to O_2_^-^, which thereby interferes with turnover of the holoenzyme^47^. Moreover, reducing agents (including NADPH) are required as cofactors for several pathway enzymes – including the Fe-S cluster enzymes but also the first committed step of the pathway catalysing the reduction and isomerization of DXP to produce MEP by DXR (Fig. 1). Redox status affects availability of these reducing agents. In plant chloroplasts these three enzymes have been identified as targets of thioredoxine (Trx)^48,49^, a member of the ferredoxin/thioredoxin system that is reduced during photosynthesis to regulate the activity of target proteins involved in a myriad of processes including the dynamic regulation of the photosynthesis or oxidative stress responses^50^. Interestingly, in this system Trx is reduced by the redox transmitter Fd-Trx-reductase (FTR) which is also an Fe-S cluster enzyme. Moreover, the electrons required for the catalytic activity of the enzyme can also be directly provided by the electron flow from photosynthesis via ferredoxin in the presence of light^50,51^.

The presence of IspG and IspH in the MEP pathway could make it particularly sensitive to the cellular oxidative environment: under oxidative stress, these enzymes may act as a bottleneck, resulting in accumulation of MEcDP, the intermediate formed immediately prior to the IspG node (Fig. 1).

### MEcDP accumulates and is exported under stress conditions

MEcDP is produced by 2*-C-*methyl-d-erythritol 2,4-cyclodiphosphate synthase (**IspF**, also known as MDS, MECPS, MECS, and MCS^26^) and is the substrate for the first MEP pathway Fe-S cluster enzyme, IspG (Fig. 1). In plants, IspG is extremely sensitive to chloroplast oxidative status; in *Arabidopsis*, oxidative stress results in reduced activity of IspG^44^ and accumulation of MEcDP^47,52^. MEcDP accumulates at high levels under oxidative stress in response to several stress inducers (including ROS, nitrosative stress, wounding, high light/temperature, pathogen attack, and heavy metals) in both plants and bacteria, and also in organisms engineered for increased MEP pathway flux^11,44,47,53–63^. Interestingly, in plants accumulating MEcDP, the existence of a second pool of this metabolite has been described based on labelling experiments^64,65^. Additionally, this MEcDP pool correlates with the accumulation of 2-*C*-methyl-d-erythritol (ME) and its C1 and C4 ME-glucosides (ME-glcs) which might be produced by the sequential ring opening, hydrolysis, and dephosphorylation followed by glycosylation^65^.

In both plants and microbes, MEcDP is exported (from the chloroplast or cell) when it accumulates^25,53,56^, although in bacteria stress-related accumulation of MEcDP does not always result in export^61^. The mechanism by which MEcDP is exported (from the chloroplast and from prokaryote cells) is poorly understood. In *E. coli* engineered for increased MEP pathway flux, MEcDP efflux is correlated with upregulation of the fosmidomycin resistance efflux pump (encoded by the *fsr* gene)^56,66^. However, knockout of the *fsr* gene only partially reduces the export rate. Moreover, this transport protein is not widely conserved across organisms that use the MEP pathway. It remains unclear how MEcDP is exported from chloroplasts^63^ and whether there are specific conserved mechanisms of MEcDP export. However, the second pool of MEcDP identified in *Arabidopsis* plants appears to be physically isolated and hence metabolically isolated from the main metabolic pathway, supporting the idea of MEcDP efflux in plants as part of a natural physiological response. This pool has been proposed to be converted into its derivates ME and ME-glcs in the cytosol according to the current knowledge on glycosiltransferases described as non-plastidial enzymes^64,65^.

### MEcDP acts as a stress signalling molecule in plants

Since accumulation of MEcDP was first reported in bacteria^62^ and suggested in plants^67^, this metabolite has been associated with functions far beyond its biosynthetic contribution to the isoprenoid building blocks IDP and DMADP. In the bacterial obligate intracellular pathogen *Chlamydia trachomatis*, accumulation of MEcDP is required for the dissociation of the histone H1-like protein (Hc1) from the DNA which causes decondensation of the nucleoid allowing the transition between the two developmental phases from the metabolically inert (extracellular infectious) form known as elementary bodies to the metabolically active (intracellular replicative) termed reticulate bodies^68^. However, how MEcDP accumulation is achieved during the metabolically inert phase or the actual molecular mechanism involving MEcDP for Hc1 dissociation is still unknown.

In plants, accumulation of MEcDP has been mostly linked to transcriptional activation of stress-related genes. Characterisation of the *Arabidopsis* *csb3* (later renamed as *clb4-3* or *hds3*), a partial loss of function mutant of IspG, showed an enhanced resistance to biotrophic pathogens as a result of a constitutive activation of plant defenses resulting in total salicylic acid (SA) accumulation and constitutive activation of several defense-related genes controlled by the SA-mediated signalling pathway^67^. The role of the MEP pathway and MEcDP was initially only suggested, but accumulation of the metabolite in the *csb3* mutant was later confirmed. A similar mutant, *ceh1* (or *hds4*) also has a partial loss of function in IspG and increased levels of MEcDP accumulation^53^. Similarly, this mutant also showed alterations in the expression levels of selected stress-responsive genes (increased HPL and ICS1 but unaffected AOS or any of the PhANG genes), increases in stress related SA, and increased resistance to biotrophic pathogens. However, there was no alteration of the transcript levels or the jasmonic acid (JA) metabolites in the JA-biosynthetic pathway. Similar results have been also reported in the *hds3* mutant, which also shows increased levels of SA concomitant with accumulation of MEcDP, but again, no interference of the JA-signalling pathway. This resulted in enhanced resistance to biotrophic pathogens (*Psuedomonas syringae*^53,67^) and aphids (*Brevicoryne brassicae*^65,67^), but absence of any effect on resistance to caterpillars (*Pieris brassicae*)^67^.

MEcDP that accumulates under stress conditions must be exported from the chloroplast to induce expression of stress-responsive nuclear-encoded plastidial proteins, suggesting that MEcDP is a retrograde signal from the chloroplast to the nucleus^53^. This behaviour has led to the proposal that the MEP pathway may act as a stress sense and response system^53^. Accumulation of MEcDP has also been shown to interact with auxin and ethylene signalling pathways in plants, influencing adaptive growth in response to stress^69,70^. Mutant *ceh1 *plants constitutively accumulating MEcDP show decreased levels of auxins concomitant with a reduction in the expression levels of YUC3 and YUC5, two members of the YUCCA family of rate-limiting enzymes involved in the biosynthesis of auxin. The mutant also shows decreased transcript levels and protein accumulation of the auxin-efflux carrier PIN1. Interestingly, plants under high light stress also show decreased levels of auxin and PIN1, but with no changes to transcript levels - suggesting a post-translational effect of MEcDP to adjust the abundance of the carrier^69^. Further work using the same mutant line identified the involvement of MEcDP in the accumulation of phyB to modulate seedling growth in continuous red light and the reduction of phytochrome interaction factors PIF4 and PIF5^70^. Under this light condition, high levels of MEcDP correlate with the previously-reported reduction of transcription levels of auxin biosynthesis and response genes. This results in an overall decrease in auxin levels, but with no significant alteration to other hormones including abscisic acid (ABA) and JA. Additionally, transcription of ethylene biosynthesis genes is also decreased, along with a significant reduction of ethylene. This suggests a coordination of red light signalling with auxin/ethylene regulation and hormone levels, where ethylene function depends on auxin signalling^70^.

Accumulation of MEcDP in *ceh1* plants triggers specific stimuli/stress related processes, some of which are directly related with accumulation of SA^71^. SA-independent stress response pathways, such as the unfolded protein response (UPR), are also triggered. UPR is a conserved eukaryotic response to stress which results in perturbation of the endoplasmic reticulum. MEcDP also induces expression of selected UPR and related downstream genes independently of SA and other general stress responses. However, protein levels of the induced genes show a partial SA-dependent accumulation, suggesting a multilayered and coordinated regulation of the UPR response. In this way, MEcDP accumulation promotes a partial induction of the UPR response, priming the system to allow higher tolerance and anticipation to ER stress. However, full induction is only achieved due to the accumulation of misfolded proteins^71^. The specifics of genetic responses to MEcDP signalling in plants have been reviewed in detail elsewhere^72–74^.

How MEcDP is actually transduced to the stress signalling pathways remains unresolved. However, bioinformatic analysis of the expression profiles of genes induced in the *ceh1* mutant, including UPR-regulatory genes, identified the rapid stress response element (RSRE) as a key motif of the general stress response (GSR) rapid transcription network. This network is constitutively active in *ceh1* plants primarily, but not exclusively, through the calcium-dependent CAMTA3 transcription factor response^75^. Additionally, high relative expression of the transcription factor MYB51, which is a major regulator of indole glucosinolates (GLS), has been reported in *hds3* plants showing an altered profile of GLS^63^. This indicates that MEcDP is potentially a direct regulator of MYB51. Recently, a component involved in the MEcDP signal transduction pathway has been identified^76^. The plastid-localised VIR3 is a member of the M41-like metalloprotease family that lacks the ATP-binding domain but contains a zinc-binding motif (required for the protein function). VIR3 accumulates in the presence of increased MEcDP levels and has been postulated to modulate the protein levels of GAPB and tAPX, which are involved in biosynthesis of GA3P and H_2_O_2_ detoxification, respectively^76^.

Despite these advances, the coordinated and complex regulatory network controlled by the accumulation of MEcDP is still not well understood. Most of the insights gained in the recent years to identify the signalling behaviour and responses to MEcDP have been obtained using mutant plants (*hds3* and *ceh1*) showing constitutive and extremely high levels of MEcDP. This is a very artificial situation and therefore, is unlikely to accurately represent story of natural MEcDP signalling and response pathways. Much is yet to be done to gain a full understanding of the mechanisms occurring under normal physiological conditions.

### MEcDP may act as an antioxidant

Studies from the early 1990’s onwards by Ostrovskii (also spelled as Ostrovskiĭ or Ostrovsky) and colleagues using MEcDP and other cyclic diphosphates suggest that they have antioxidant properties^11,60,77^ and can protect DNA from degradation by H_2_O_2_ in the presence of ferrous ions, possibly by complexing with said ions^60^. These publications are in Russian, which limited broader community access to this research. Their later publications in English reviewed and further presented the behaviour of MEcDP as an antioxidant (or ‘antistressor’)^55,62^. However, the MEcDP molecule does not have classical features usually associated with an antioxidant molecule, and is apparently quite stable in vivo^78,79^. A caveat is that in vitro it has been observed that MEcDP isolates are stable at pH 4-5 but undergo spontaneous transformation into several products at neutral and alkaline pH^53^. Its potential antioxidant role remains controversial in the field^80^.

### Intracellular levels of MEcDP are extremely tightly regulated

Ex vivo experiments suggest that IspF (also known as MDS or YgbB; MEcDP synthase) is subject to an unusual positive feed-forward control by the upstream metabolite 2*-C-*methyl-d-erythritol-4-phosphate (MEP; Fig. 1)^81^. This means that if MEP accumulates, IspF activity increases, potentially providing a metabolic ‘pull’ in vivo. In addition, downstream prenyl diphosphates can bind to IspF, and FDP can inhibit the IspF-MEP complex, providing a negative feedback mechanism (Fig. 1)^81,82^. This means that as MEP accumulates upstream, flux to MEcDP increases, whereas if FDP accumulates downstream, flux decreases. This degree of regulatory sophistication around the MEcDP node points to a particularly critical physiological role of MEcDP.

### The MEP pathway produces, and is controlled by, antioxidant metabolites, stress hormones, and regulatory molecules

The MEP pathway produces many metabolites that play roles in redox balance and oxidative stress (e.g. tocopherols, carotenoids, hormones). Isoprene, a volatile C_5_ isoprenoid, protects plants from oxidative stress through a mechanism yet to be uncovered which results in increased levels of antioxidants and inhibition of ROS accumulation^83^, and it has been proposed that volatile isoprenoids play a generic stress-protection role^16^. ROS are reduced, and antioxidants increased, in transgenic plants producing isoprene in the absence of oxidative stress, and isoprene-emitting plants under oxidative stress respond much more quickly that non-emitting plants^83^. This suggests that emitting plants are primed for fast responses to oxidative stress, in turn suggesting a role in influencing ROS response networks. However, the mechanism of protection by isoprene is not yet fully elucidated.

In *Salvia miltiorrhiza* (red sage), the stress-response hormones ABA (an isoprenoid product) and methyl jasmonate (MJ), as well as exogenous chemical stressor treatment (polyethylene glycol), increase DXS enzyme activity through upregulation of *dxs* transcription^84^. These observations suggest that upstream MEP pathway flux increases as a general response to stress. Conversely, flux through DXS decreases in response to herbivory^52^ in a complex response partly driven by herbivory-induced production of ROS. Herbivory and mechanical wounding downregulate *dxs* transcription^52,85^, but DXS enzyme activity is also competitively inhibited by accumulation of β-cyclocitral (βCC), which is produced through photo-oxidation of the isoprenoid pigment β-carotene by ^1^O_2_ and has been described as a stress signal that triggers transcriptional responses associated with increased tolerance to photooxidative stress^86^. This oxidation occurs due to an increase in ROS and particularly to ^1^O_2_, concomitant with an increased pool of MEcDP and the export of this metabolite from the plastid. The increase in MEcDP occurs despite the downregulation of DXS most likely because IspG activity is also downregulated by ROS accumulation which implies that the decrease in flux through IspG is greater than the decrease in flux through DXS under these circumstances.

## Implications, observations, and questions about the MEP pathway, MEcDP, IspG, IspH, and oxidative stress

Together, the above observations imply an important role for the MEP pathway in stress sensing, signalling, and protection, which is conserved to some extent across kingdoms. This role goes beyond the well-recognised activities of hormones and protective molecules made downstream of prenyl phosphate metabolism. MEcDP and the two terminal Fe-S cluster enzymes are key components of this newly-recognised sense and signalling behaviour.

### Exploring the significance of non-biosynthetic roles for the MEP pathway

A major question in the field of isoprenoid biochemistry is why plants continue to harbour two complete, nonredundant isoprenoid pathways^87^. It can probably be presumed that the MVA pathway evolved first, since archaebacteria precede eubacteria. The presence of Fe-S clusters in the MEP pathway suggests that it evolved prior to the Great Oxygenation Event. Eukaryotes obtained the MEP pathway along with the organisms that were endocytosed and ultimately developed into organelles. In most cases, this has been reduced to stand-alone prenyl phosphate metabolism (e.g. in the mitochondria), but in plant plastids it is maintained as a complete pathway. Regulatory considerations as well as ecological and functional activities of different pathway products are likely to form part of the explanation for the nonredundant coexistence of the MVA and MEP pathway in plants^87^ and also for prenyl phosphate metabolism in non-chloroplast plastids.

Very interestingly, the archaeal MVA pathway uses an Fe-S cluster enzyme, phosphomevalonate dehydratase, whereas eukaryotes use an alternative non-Fe-S cluster enzyme at that step in the pathway^30^. This is fascinating because it indicates that the eukaryotic pathway has evolved away from oxygen sensitivity conferred by the Fe-S cluster enzymes, and as such is far more resilient to oxidative stress. The presence of a stress-sensing function explains why oxygen-sensitive chemistry has been maintained in the MEP pathway, as opposed to replacement with the less sensitive, less complex, and less expensive chemistry. It may also contribute to the explanation for the retention of two nonredundant isoprenoid pathways in plants. This also suggests that the oxygen sensitivity of the pathway may be critical for its function.

**Fig. 3:**
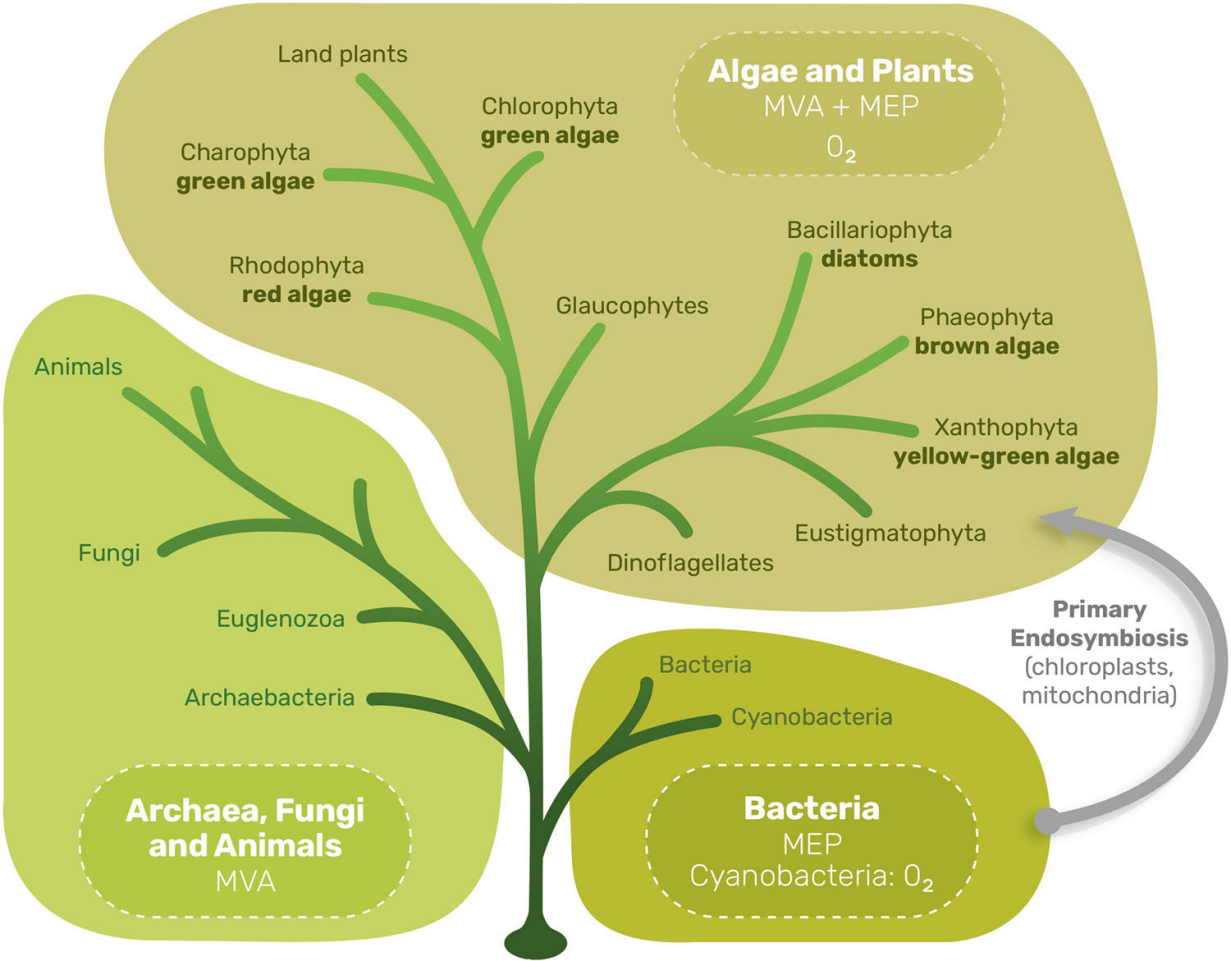
Simplified tree with emphasis on evolutionary groupings with the MEP and MVA pathways. Very roughly (with notable exceptions), the MVA isoprenoid pathway is found in eukarya and archaea while the MEP pathway is found in bacteria. Plants and algae have both; the MEP pathway is maintained in chloroplasts or chloroplast-like organelles, along with the machinery for oxygenic photosynthesis (groups that perform oxygenic photosynthesis are labelled with ‘O_2_’). Primary endosymbiosis is shown with an arrow; this ultimately resulted in mitochondria (from proteobacteria-like organisms) and chloroplasts (from cyanobacteria-like organisms) in glaucophytes, green algae, red algae, and eventually, higher plants. Other algal groups are thought to have arisen from endosymbiosis of one eukaryotic alga into another eukaryote (secondary and tertiary endosymbiosis). Limited prenyl phosphate metabolism, but not a MEP pathway, is found in mitochondria. Image edited and adapted using work by Govindjee and Shevela^109^, Hallman^110^, Blankenship^111^, and Blankenship et al.^112^.

The presence of the (full or partial) MEP pathway in some microbes that produce isoprenoids via the MVA pathway^88^ is additional evidence that there are evolutionary pressures other than those related to biosynthesis of isoprenoids on the MEP pathway. The MEP pathway is always present where oxygenic photosynthesis is present, but clearly evolved before photosynthesis, as it is present in the eubacterial clade (Fig. 3). In most cases where both the MEP and MVA pathway are present, the MEP pathway is maintained in a chloroplast or chloroplast-like structure (Fig. 3).

The *Listeria* case described above is interesting, as listeria are eubacteria but have only a partial MEP pathway, instead using the MVA pathway for isoprenoid biosynthesis^88–90^. These bacteria are intracellular parasites in mammals, an unusual niche that places them in direct contact with mammalian host cell metabolism. This may have provided an opportunity for horizontal gene transfer followed by partial loss of the MEP pathway; the signalling and/or antioxidant behaviour of MEcDP obviously retained an evolutionary benefit in this environment in this scenario. Interestingly, some eukaryotic intracellular pathogens, including the Apicomplexan protozoan pathogens *Toxoplasma gondii* (the causal agent of toxoplasmosis) and *Plasmodium* spp. (the causal agent of malaria), exclusively use the MEP pathway for isoprenoid biosynthesis. These observations support the hypothesis that MEcDP is important in such intracellular interactions, potentially though its antioxidant behaviour (interfering with cellular ROS responses to infection) or through some signalling behaviour. The MEP pathway is required for virulence of *Listeria monocytogenes* in mice^88^ and IspF specifically has a role in oxidative stress and virulence in mice^91^. While the mechanism(s) remain to be elucidated, these observations suggest that MEcDP plays a role in animal infection as well as in plant infection.

### The role of oxygen sensitivity of IspG and IspH

Fe-S cluster chemistry has a deep pre-oxic evolutionary history; oxygen sensitivity was essentially irrelevant when this chemistry evolved. Conversion of the Earth’s environment to an oxidative one provided an evolutionary pressure for evolution away from oxygen sensitivity where possible, and that may explain why the eukaryotic MVA pathway lost its Fe-S cluster enzymes. Maintenance of the oxygen sensitivity of the MEP pathway enzymes suggests that there is value in having that oxygen sensitivity. Here, we suggest that alternative roles in sensing oxidative stress provided that evolutionary pressure to maintain IspG and possibly also IspH as oxygen-sensitive Fe-S cluster enzymes.

The sensitivity of the Fe-S cluster has been exploited by evolution as a sensor to detect oxidative stress in other contexts. For example, the model organism *E. coli* can survive in both aerobic and anaerobic environments, but it needs a mechanism to sense the oxygen status of its environment in order to trigger the appropriate metabolic response^92^. The fumarate and nitrate reduction transcription regulator (FNR) enzyme of *E. coli* utilises a 4Fe-4S cluster to form a transcriptionally active homodimer, which dissociates into a 2Fe-2S cluster in the presence of oxygen to yield an inactive monomeric variant. This switch allows the bacteria to change between anaerobic and aerobic metabolism according to O_2_ levels.

### Why are there two adjacent oxygen-sensitive Fe-S cluster enzymes?

IspG alone should be sufficient to initiate a stress sensing/response mechanism through accumulation of MEcDP in response to oxidative stress. So, what is the role of the maintained oxygen sensitivity of IspH? Perhaps it is simply that there was insufficient evolutionary pressure for this node to evolve away from oxygen sensitivity in the presence of a necessary maintenance of sensitivity of IspG. We recently identified that IspH proteins from different organisms have an evolved IDP:DMADP production ratio that likely relates to the metabolic requirements of the host organisms for different chain-length isoprenoids^93^; perhaps this ratio is influenced by the oxidative environment in the cell, potentially allowing production of different chain-length isoprenoids under different conditions. IspH is encoded by multiple non-redundant genes in certain plant species^93,94^; harbouring paralogs that produce varying ratios of DMADP/IDP could provide these species with a means to react to oxidative stress. Thus, there could be evolutionary pressure to maintain IspH. The terminal enzyme of the MVA pathway produces only IDP, which is then isomerised to make DMADP by isopentenyl disphosphate isomerase (IDI)^95^; there are no production ratio constraints on its chemistry, possibly making it more flexible to different evolutionary pressures.

Another possibility is that differential sensitivity of IspG and IspH to different ROS is an important part of a nuanced response to oxidative stress. Interestingly, there is some evidence that IspH has evolved to adapt to different O_2_ levels in different environments^96^. Rao et al. have identified four different classes of IspH correlating to different organisms: aerobic bacteria, anaerobic bacteria, plants, and parasites^96^. These classes have different N- and C-terminal regions that specifically modulate sensitivity of the enzymes to oxidation based on the presence of protective aromatic residues. IspH proteins from plants and parasites have an N-terminal region containing a tyrosine amino acid that is postulated to protect the cluster. IspH proteins from aerobic bacteria (including *E. coli*) have an additional C-terminal region of ~25 amino acids that provides extra protection to the enzyme, while IspH proteins from anaerobic bacteria do not have this peptide^96^. On the other hand, IspG seems to show lower stability to some oxidants than IspH, with a rapid degradation of the cluster in its open (absence of substrate binding) form^47,58,96,97^.

This potential differential inhibition might explain the formation and accumulation of two HMBDP-derived hemiterpenoid glycosides (HTG) (isomers 1-glycoside and 4-glycoside of (2*E*)-2-methylbut-2-en-1,4-diol glycoside) in plant leaves under root NO_3_^−^ deprivation, root wounding, or root exposition to oxidative stress upon peroxide treatment. Phenylpropanoids also accumulate under these conditions, indicating a broader stress response to these treatments. Other stresses, such as cold, saline, or osmotic stress do not induce the formation of HTGs on *Arabidopsis* leaves suggesting a specific response to the accumulation of ROS. Exogenous HMBDP or diols are converted to HTGs independently of the nutrient status of the plant suggesting that production of these compounds is not dependent of the diol formation and glycosylation steps which remain fully active in all nutrient conditions. The accumulation of HMBDP (a metabolite that usually do not accumulate under normal growing conditions) appears to be required to divert the MEP pathway to the formation of the HTGs which has been postulated to happen by restricting the flow through IspH. The implication of a specific inhibitor controlling the activity of IspH cannot be excluded but alternatively a differential sensitivity of IspH to specific stress conditions could also explain the accumulation of HMBDP to allow the production of the HTGs^98^. The formation of HTGs has also been described from MEcDP in MEcDP-accumulating mutant plants (*hds3*) but also, to a much lower extent, in wild-type plants, suggesting that the formation of these compounds is indeed a natural physiological process. MEcDP undergoes ring opening and dephosphorylation generating ME which is then glycosylated in positions 1 and four to generate 2-*C*-methyl-d-erythritol-*O*-1-β-d-glucopyranoside (ME-1-Glc) and 2-*C*-methyl-d-erythritol-*O*-4-β-d-glucopyranoside (ME-4-Glc) respectively. The enzymatic activities involved in these transformations are currently unknown, but the formation of these ME-derived HTGs happens thorough a physically isolated second pool of MEcDP most likely in the cytosol in accordance with the absence of glycosidases in the chloroplast. The biological function of the HMBDP/MEcDP-derived HTGs is unknown but glycosylation has been previously described as a deactivation mechanism of some phytohormones suggesting that these metabolites might also be deactivated forms of active signals^65^.

These results indicate a potential differential sensitivity to oxidative stress allowing different capacities of the two enzymes to respond to oxidative stress and therefore to control accumulation of the intermediates MEcDP, HMBDP, and/or IDP and DMADP. This would in turn allow fine tuning of the pathway behaviour according to the stress conditions and response requirements. As noted above, the ratios of IDP and DMADP determine the potential of the pathway to deliver different chain-length isoprenoids^93^; this provides a potential mechanism to affect cellular levels of antioxidant metabolites downstream of prenyl phosphate metabolism.

All of these musings are of course highly speculative and require experimental testing to further explore them.

### The role of MEcDP in stress responses

Many isoprenoid stress response metabolites, such as tocopherols and carotenoids, are produced constitutively and maintained in pools to support stress responses. Others, such as isoprene, are produced de novo. It appears that MEcDP also has a role as a de novo stress response metabolite and may act both as a signalling molecule and possibly as an antioxidant – although this needs to be verified. Accumulation of MEcDP under stress in such a broad range of clades also indicates that the function of MEcDP as a stress signalling molecule has an extremely deep evolutionary history.

If MEcDP behaves as an antioxidant in vivo, it could potentially limit oxidative damage to oxidation-sensitive enzymes in the pathway and elsewhere in metabolism, as well as providing general cellular protection from intra- and extra-cellular oxidative damage^60,99^. Accumulation of MEcDP under stress may therefore be doubly advantageous, in that it could help to deplete ROS directly as well as acting as an indirect signalling molecule via immediate initiation of stress response pathways which, in the longer term, drive physiological and phenotypic responses via growth modifications. However, this is a poorly understood area of potential impact for MEcDP, and requires further research to examine many different questions, for example: does MEcDP have antioxidant behaviour? What are the accumulation patterns, and how do they differ in different organisms? More generally, the function of MEcDP in microbes (beyond its role as a MEP pathway intermediate) requires further examination. Ostrovskii and colleagues first proposed a role for MEcDP in the response of nocardioform bacteria to oxidative stress in the mid-90’s^11,77^ (Russian language publications), around the same time that the MEP pathway was discovered. In the mid-2000s MEcDP was shown to interact with chlamydial histone-like proteins, disrupting DNA association and allowing for transcriptional activation^68,100^. Interestingly, a truncated MEP pathway is retained in some organisms that also harbour an MVA pathway. *Listeria innocua* uses the MVA pathway for isoprenoid production, but also harbours a partial MEP pathway, up to MEcDP production^88^. When the genes for the missing steps are introduced, full pathway functionality is restored. Clearly, retention of MEcDP biosynthesis serves some important purpose other than production of isoprenoids in *L. innocua*. Alternatively, these organisms might have non-canonical enzymes that perform the same function but do not share homology with the canonical pathway enzymes – similarly to the DXR-like (DRL) enzymes found in *Brucella abortus* and other organisms, which performs the same reaction as DXR^101^. *L. monocytogenes*, a closely related pathogenic organism, harbours functional versions of both pathways, however the MEP pathway alone cannot sustain growth under non-pathogenic conditions^89,90^. This suggests that it, at least, does not have enzymes that can replace IspG/H functionality. Finally, the *L. monocytogenes* virulence regulator Prfa has been linked to increased resistance to oxidative stress through the *ispF* gene (among others)^91^. It is tempting to speculate that MEcDP plays a direct role in pathogenesis, perhaps neutralising ROS produced by the host cell under attack. Further investigations may reveal a more direct role of MEcDP in this response.

### The unusual chemistry of MEcDP and its implications

It is worth considering the chemistry of MEcDP with respect to its biological roles. MEcDP is an unusual molecule that is cyclised through the diphosphate moiety (Fig. 1), which is a very uncommon structure to find in biology. This chemistry likely confers relative stability on the molecule (it is stable in solution at room temperature for weeks and is not dephosphorylated by alkaline phosphatase^78^), a property which is desirable for a signalling molecule with intra-organelle transport requirements (plants) and potential extracellular functions (microbes). In contrast, prenyl diphosphates, which are linear molecules with the phosphates at one terminus, are quite labile; they easily lose their phosphate groups when stored (even at −80 °C) and in common laboratory manipulations^102^. A wide variety of cellular phosphatases, including many non-specific ones, act to cleave terminal phosphate bonds and recycle phosphate in vivo. Perhaps the unusual cyclic chemistry of MEcDP facilitates its role as a signalling molecule by preventing phosphatase access to phosphate bonds.

IspG must accommodate the ring structure in its active site and break this cyclic diphosphate chemistry to re-integrate MEcDP into MEP pathway metabolism. This enzyme falls into two classes based on number of domains; two-domain IspG is found in bacteria, while three-domain IspG is found in plants and Apicomplexa^103^. In two-domain IspG, the Fe-S cluster binding site is ~4 nm away from the substrate binding site (too far to initiate the reaction) and must dimerise, creating two active sites per dimer^36^. Three-domain IspG has an inserted domain expected to function structurally, possibly providing enough flexibility for monomeric catalysis^103^. Interestingly, ring opening of MEcDP does not require electron transfer; it occurs in equilibrium with the closed form once bound (the ring is permanently opened after electron transfer)^79^. As isotopic exchange experiments that led to this discovery were conducted with two-domain *E. coli* IspG, in addition to the lack of three-domain crystal structures, it is uncertain whether plant IspG interacts with MEcDP in the same manner.

### A plethora of mechanisms provides an opportunity for the MEP pathway to perform different metabolic functions and deliver nuanced responses to different oxidative stresses

Here we have examined multiple different potential and actual response mechanisms, ranging from antioxidant behaviour to differential oxygen sensitivity and extremely complex metabolic regulation responses. Why does one pathway have so many different nuances apparently related to oxidative stress responses? Here we will explore these nuances and why they might occur.

Firstly, not all ROS interact with Fe-S clusters equally. O_2_ is only weakly oxidising compared to ^1^O_2_, H_2_O_2,_ O_2_^-^, and OH**·**^104^, however due to its electronic structure and outer sphere electron transfer mechanism it can oxidise from a distance (i.e. no bond forming-breaking is required for electron transfer)^105^. Rates of oxidative degradation also vary between ROS and enzyme structures; Fe-S cluster dehydratases are oxidised by H_2_O_2_ and O_2_^-^ by ~10^4^ and ~10^6 ^M^−1^ s^−1^ respectively^106^, while O_2_ oxidises the 4Fe-4S cluster of *E. coli* FNR at a rate of 250 M^−1^ s^−1^^107^. This provides a potential mechanism for more nuanced responses to different oxidative stresses, and would be an interesting subject to pursue further for IspG and IspH. One approach to examine this might be electron paramagnetic resonance (EPR), which might detect the presence of 4Fe-4S and/or 2Fe-2S clusters: both types may be present but in changing concentrations depending on the level of oxygen exposure. EPR is limited to paramagnetic species, and thus dimagnetic clusters such as 4Fe-4S^2+^ would be EPR-silent. Native mass spectrometry has more recently been used to detect cluster variants, as it is an extremely sensitive technique that does not have constraints on oxidation state^108^. Such investigations might also provide clues as to why IspH remains oxygen-sensitive.

It is also tempting to speculate that, if MEcDP does indeed behave as an antioxidant (noting the caveat previously mentioned), this MEcDP may allow discrimination between different responses to stress. Even with a weak antioxidant behaviour, a local accumulation of MEcDP around IspG might act as a devoted protectant of IspG. This way the cell could prevent triggering responses mediated by MEcDP but still allow other stress-response mechanisms to happen. This might also provide a mechanism for differentiating between the oxidative stress response functions of IspG and IspH. As discussed above, differential sensitivity of IspG and IspH to ROS may be an important part of a nuanced response to oxidative stress, with MEcDP protection forming part of the response equation.

In addition, Ostrovskii and colleagues observed that isoprenoid biosynthesis and MEcDP biosynthesis in bacteria are ‘alternative processes’^55^, suggesting that accumulation of MEcDP in the presence of oxidative stress prevents downstream biosynthesis of isoprenoids. It is unknown how MEcDP accumulation affects downstream products in plants, however, over time it would be expected that accumulation and export of MEcDP would impact on downstream products including antioxidants such as carotenoids. It may therefore functionally replace downstream antioxidants (although, this would be regardless of whether its activity is as an antioxidant or as a signalling molecule to trigger other antioxidant responses). We have few other clues as to how MEcDP might interact with global cellular stress responses. However, examples such as the signalling role of carotenoid-derived metabolites (βCC) generated by oxidation of β-carotene in the presence of ^1^O_2 _causing a reduction of the MEP pathway flux through transcript and activity reduction of DXS but showing accumulation of MEcDP in response to herbivory already suggest the complex interactions and balance between complementary responses and the ability to coordinate specific responses under different kinds of stress.

Finally, as discussed above, the MEP pathway is heavily regulated at almost every node in the pathway, and at every possible level – transcriptional, post-transcriptional, post-translational, and allosteric. This suggests a highly nuanced pathway behaviour. The regulatory mechanisms so far identified (Fig. 1) are probably the tip of the proverbial iceberg.

Of course, the mechanisms described above all occur on different timelines, have different magnitudes, and have different metabolic repercussions. Sometimes, as in the case of ABA up-regulation of DXS^84^ and down-regulation of the MEP pathway in response to herbivory^52^, responses occur in apparently opposing and counter-intuitive ways. The timing, magnitude, and consequences of these responses probably reflects a plethora of mechanisms suitable for a wide variety of physiological stress situations, including immediate and then delayed responses to stress. This in turn suggests that the MEP pathway represents a highly complex and evolutionary refined system for nuanced responses to a wide variety of stresses.

How might this look? This question leads to highly speculative, but potentially testable, theories about how biology might manage a nuanced approach to oxidative stress responses at the IspG node. For example: in oxidative conditions, IspG loses activity due to inactivation of the Fe-S cluster; this in turn promotes the accumulation of MEcDP. MEcDP might alleviate the stress on IspG due to its potential antioxidant activity. However, if the level of ROS is too high or the stress lasts too long, then IspG activity is so low that MEcDP accumulates at very high levels and finally is exported to trigger a more complete response to the stress. This scenario is envisioned for the chloroplast/nuclear signalling, but what about bacteria, which also export MEcDP? Perhaps, MEcDP simply has an antioxidant role and is non-selectively exported when it accumulates at very high levels. Conversely, MEcDP might play a role in signalling oxidative stress to other bacterial cells. Such behaviour might then have evolved into the much more complex retrograde signalling role observed in plants.

Complex regulatory control and the presence of many different mechanistic behaviours likely allows the MEP pathway to perform a variety of different functions at different metabolic levels, delivering multiple highly nuanced services for the host cell.

## Future directions

The assembled evidence to date demonstrates a clear role for the MEP pathway, and in particular the intermediate MEcDP, in oxidative stress sensing, signalling, and responses. While the field has advanced significantly in recent years and some mechanisms are at least partially elucidated (e.g. the role of MEcDP as a retrograde signaller), many questions about the MEP pathway and its role in the cellular oxidative stress response system remain. These include questions about the evolution and adaptation of the MEP pathway in different kingdoms; for example: What drove evolution of the MEP pathway in an anoxic environment, and in the context of the already-existing MVA pathway? Exactly what role does the MEP pathway/MEcDP play in pathogen infection in animals, and what are the mechanisms of this role? What other mechanisms come into play in the MEP pathway/ROS response system across different kingdoms?

There is also much left to elucidate about the oxygen response mechanisms; for example: How do different ROS affect IspG and IspH? Is there differential regulation or O_2_ sensitivity at the IspG and IspH nodes that drives differential accumulation of MEcDP and HMBDP, and what are the repercussions of such a differential response? Does MEcDP have antioxidant activity? If so, how effective is MEcDP in vivo as a cellular antioxidant, and how does it interact with other cellular antioxidant and stress response mechanisms? To what extent does MEcDP accumulate during stress, and how much accumulates before it starts being exported? And does the idea of MEcDP having an antioxidant role conflict with the fact that it gets exported from the stressed environment? Does oxidation of MEcDP affect its function as a signalling molecule?

The signalling behaviour and role of MEcDP more broadly is also a source of significant uncertainty. Why do some bacteria export MEcDP and under what conditions? Data to date has been gathered from engineered bacteria with high pathway fluxes, from attacking pathogens, or from conditions with uncertain physiological relevance, and it is unclear how universal this export is. Does it behave as a signalling molecule to other bacteria, similar to its retrograde signalling role in plants? How does MEcDP get out of and into cells/cellular compartments? As noted above, there is some evidence that the fsr transporter can translocate MEcDP^56,66^, but it is unlikely to be the only transporter and unlikely even to be the most important transporter. A chloroplast transporter has not been identified. Similarly, no receptor for MEcDP has been identified. Identification of receptors will be required to fully characterise the signalling cascade that we presume is initiated by MEcDP.

Finally, there are more tangential questions about the MEP pathway that may be related to its oxygen response behaviour, for example: What enzyme characteristics are responsible for the control of DMADP/IDP ratio in IspH? Can this ratio be modified by cellular conditions, and if so, what effect does this have on downstream metabolites? Does this alter the ratios of antioxidants in response to stress?

Answering these questions will take significant investment and likely lead to further fascinating insights into this remarkable pathway. As we progress, we may find that some of the answers can be exploited for interesting biotechnological applications, for example: industrial production of useful isoprenoids, management of plant oxidative stress responses (which will become increasingly important in climate change conditions), control of plant and animal pathogen infection processes, development of antimicrobials, and potentially even management of microbiome behaviour.
